# Raman Spectroscopic Study of Radioresistant Oral Cancer Sublines Established by Fractionated Ionizing Radiation

**DOI:** 10.1371/journal.pone.0097777

**Published:** 2014-05-19

**Authors:** Mohd Yasser, Rubina Shaikh, Murali Krishna Chilakapati, Tanuja Teni

**Affiliations:** 1 KS-121, Teni Laboratory, ACTREC, Tata Memorial Centre, Kharghar-Node, Navi Mumbai, India; 2 KS-04, Chilakapati Laboratory, ACTREC, Tata Memorial Centre, Kharghar-Node, Navi Mumbai, India; Technische Universitaet Muenchen, Germany

## Abstract

Radiotherapy is an important treatment modality for oral cancer. However, development of radioresistance is a major hurdle in the efficacy of radiotherapy in oral cancer patients. Identifying predictors of radioresistance is a challenging task and has met with little success. The aim of the present study was to explore the differential spectral profiles of the established radioresistant sublines and parental oral cancer cell lines by Raman spectroscopy. We have established radioresistant sublines namely, 50Gy-UPCI:SCC029B and 70Gy-UPCI:SCC029B from its parental UPCI:SCC029B cell line, by using clinically admissible 2Gy fractionated ionizing radiation (FIR). The developed radioresistant character was validated by clonogenic cell survival assay and known radioresistance-related protein markers like Mcl-1, Bcl-2, Cox-2 and Survivin. Altered cellular morphology with significant increase (p<0.001) in the number of filopodia in radioresistant cells with respect to parental cells was observed. The Raman spectra of parental UPCI:SCC029B, 50Gy-UPCI:SCC029B and 70Gy-UPCI:SCC029B cells were acquired and spectral features indicate possible differences in biomolecules like proteins, lipids and nucleic acids. Principal component analysis (PCA) provided three clusters corresponding to radioresistant 50Gy, 70Gy-UPCI:SCC029B sublines and parental UPCI:SCC029B cell line with minor overlap, which suggest altered molecular profile acquired by the radioresistant cells due to multiple doses of irradiation. The findings of this study support the potential of Raman spectroscopy in prediction of radioresistance and possibly contribute to better prognosis of oral cancer.

## Introduction

Oral cancer is the sixth most common cancer worldwide [1]. In India, extensive tobacco usage in various forms makes it the leading type of cancer in males and third most common cancer in females [2], [3]. Also, prevalence of oral buccal mucosa cancer type is high in the Indian subcontinent [4]. The treatment modalities of oral cancer are based on various factors including disease stage, access to the oral site, age and physical status of patient. Although surgery is choice of treatment in early stages; radiotherapy holds an important place either alone or as an adjuvant with chemotherapy [5], [6]. Standard radiotherapy protocol involves daily exposure of 2Gy fraction dose for few weeks, where patients receive a cumulative dose of 50Gy to 70Gy during the radiotherapy course [7], [8], [9]. Fractionated radiotherapy kills fast dividing tumour cell population with decreased effects on surrounding normal tissues. Thus this method provides time for normal cells to repopulate and recover while diminishing tumour cells that have aberrantly activated signal transduction pathways [10], [11]. However, sometimes tumour recurs with an acquired radioresistant phenotype posing as an obstruction towards the efficacy of radiotherapy. In order to make radiotherapy more effective; it is important to explore the radioresistant phenotype in cancer cells. Association of several proteins such as p53 [12], Cox-2 [13], Ras [14], pAKT [15], MDM2 [16], Clusterin [17], Survivin [18], Bcl-2 [19] and Mcl-1 [20] with radioresistance have been reported earlier. However, so far there is no available tool that can predict radiotherapy response in oral cancer patients leading towards better treatment.

Biomedical application of optical spectroscopic techniques like Fluorescence, Fourier transfer infra-red (FTIR), Diffused reflectance and Raman spectroscopy (RS) for classification of different pathological conditions and cancer detection has already been reported [21]–[24]. Among these techniques, RS has added advantages like it is label free, sensitive to biochemical variations, applicable to *in vitro* and *in vivo* conditions, has minimum interference from water and provides molecular fingerprints [25]–[27]. Our previous studies have demonstrated the efficacy of RS in classifying healthy, premalignant and malignant lesions of oral submucosa [28]–[29]; classification of the normal and abnormal exfoliated cells [30] and in the prediction of tumour response towards concurrent chemo-radiotherapy in cervical cancers [31]. We have shown the potential of RS in identifying early transformation changes in oral buccal mucosa [32], its feasibility in detecting asthma and determining treatment response through serum in asthma patients [33], in classifying normal and oral cancer serum [34] and in identifying multidrug resistance phenotype in human leukemia [35] and uterine sarcoma cell lines [36].

**Figure 1:**
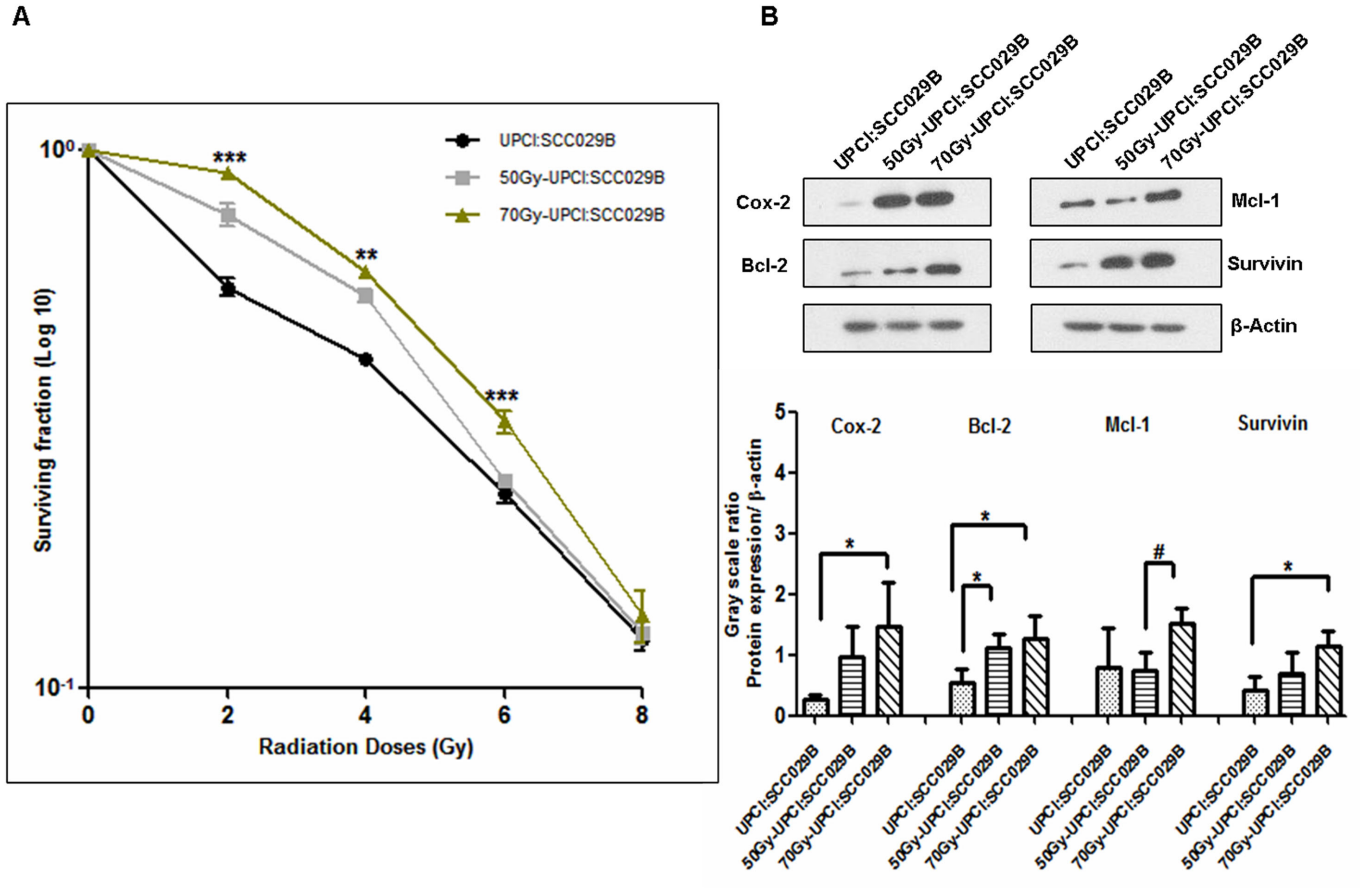
Radiosensitivity of parental and established radioresistant sublines. (A) Clonogenic cell survival curve. UPCI:SCC029B parental cells, 50Gy-UPCI:SCC029B intermediate radioresistant cells and 70Gy-UPCI:SCC029B final radioresistant cells. Data is represented as percentage survival of cells with mean (±SD) of three independent experiments. One-way ANOVA statistical analysis was performed on surviving fractions at the given doses, p<0.01 & 0.001. (B) Expression of radioresistance markers in radioresistant oral sublines. Western blotting for Cox-2, Bcl-2, Mcl-1 and Survivin proteins; β-Actin is used as loading control. Densitometry analysis of western blots from three separate experiments is shown below, bars represent mean ± s.d. * represent protein expression compared to parental cell line, while # represent protein expression compared between 50Gy-UPCI:SCC029B and 70Gy-UPCI:SCC029B sublines (p<0.05).

RS studies related to radiation induced biochemical changes in prostate, lung and breast cancer cell lines irradiated with radiation doses between 15 and 50Gy are reported [37], [38]. These studies were carried out at single doses of radiation that aimed to investigate the *in vitro* radiation response on human cancer cell lines. On the other hand, we carried out the present study, taking advantage of continuous low dose fractionated irradiation routinely used as standard radiotherapy protocol in clinics for oral cancer treatment. Our aim was to develop *in vitro* radioresistance character in the cell line over a period of time and then explore the feasibility of Raman spectroscopy to categorize the acquired trait from its parental untreated cells. We have established radioresistant oral cancer sublines of buccal mucosa origin by clinical implementable 2Gy fractionated radiation dose. After establishing the sublines, their radioresistant character was evaluated by clonogenic cell survival assay and Raman spectral profiles were obtained by RS. To the best of our knowledge, we are first to report the utility of RS in acquired radioresistant oral cancer sublines established from parental oral cancer cell line by clinically administered fractionated ionizing radiation.

## Materials and Methods

### Establishment and Characterization of Radioresistant Cell Lines

#### a) Cell culture and establishment of radioresistant sublines by gamma radiation treatment

UPCI:SCC029B, human oral buccal mucosa carcinoma cell line was kindly provided by Dr. Susanne M. Gollin, University of Pittsburgh, USA [39]. Cells were maintained in Dulbecco's Modified Eagle Medium (DMEM, Gibco) supplemented with 10% heat inactivated fetal bovine serum (FBS, Sigma-Aldrich) and antibiotics (100 U/ml penicillin and 100 µg/ml streptomycin). Cells were maintained at 37°C in 5% CO^2^ humidified atmosphere.

**Figure 2:**
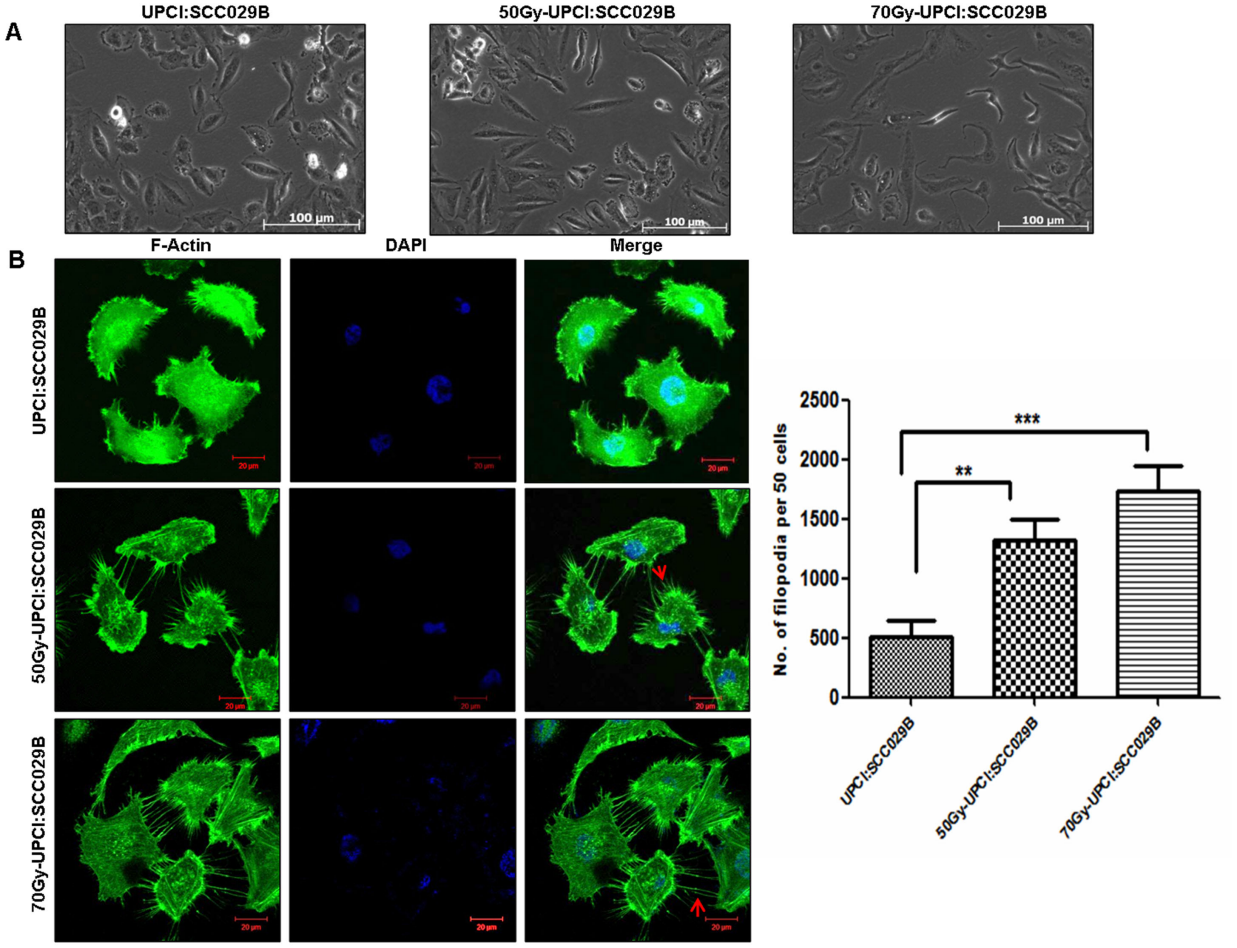
Fractionated irradiation leads to change in cell morphology. (A) Cell morphology analysis. Parental UPCI:SCC029B cells, radioresistant 50Gy-UPCI:SCC029B and 70Gy-UPCI:SCC029B cells. Scale bars: 100 µm. (B) Confocal images of filamentous-Actin stained with Alexa Fluor-488 phalloidin, cells were counter stained with DAPI. Scale bars: 20 µm. Arrow indicates filopodia as fine cell surface extensions. Analysis based on 50 cells per population with mean and standard deviation of three independent experiments, plotted at below right side (p<0.01, 0.001).

To generate radioresistant sublines, UPCI:SCC029B cells (1×10^6^ cells) were seeded in 100 mm culture plates (BD-Biosciences) containing complete media. Cells were grown in standard condition and were irradiated with 2Gy of ionizing radiation using ^6^°Co-γ Linear Accelerator (Bhabhatron-2, ACTREC, Tata Memorial Centre) at 60% confluency. Immediately after irradiation the culture medium was renewed and cells were placed in incubator till they reached 90% confluency. Cells were then trypsinized, counted and passage into new culture plates. The cells were treated again with 2Gy of ionizing radiation at about 60% confluency. This procedure was repeated 25 times for generation of intermediate 50Gy-UPCI:SCC029B radioresistant subline and further continued upto 35 times over a period of 5 to 6 months till generation of final 70Gy-UPCI:SCC029B radioresistant subline.

**Figure 3:**
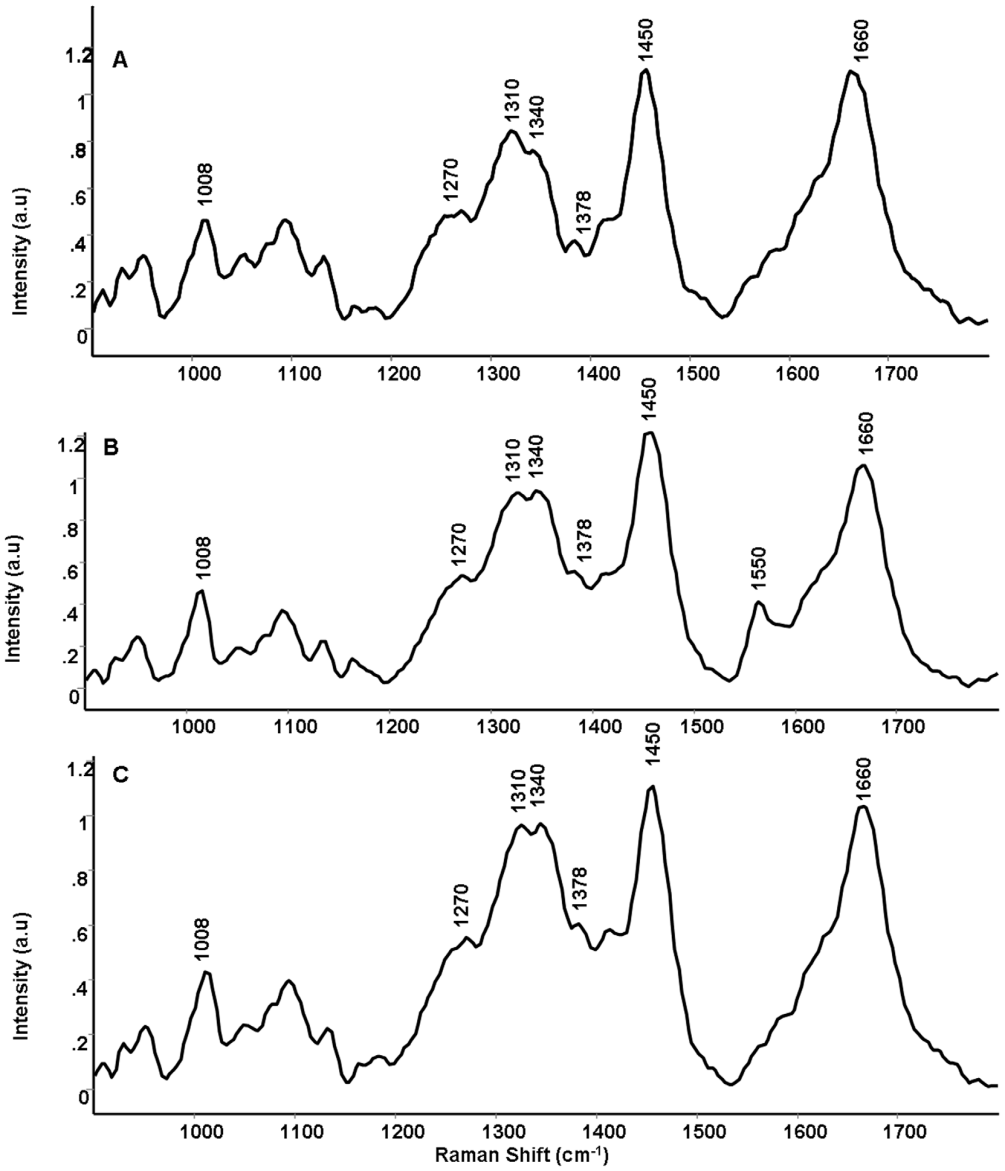
Mean Raman spectra of parental and radioresistant sublines. (A) Parental UPCI:SCC029B cell line (B) 50Gy-UPCI:SCC029B subline (C) 70Gy-UPCI:SCC029B subline.

#### b) Clonogenic cell survival assay

Briefly, known number of both the parental and radioresistant cells of UPCI:SCC029B were seeded in 100 mm culture plates and kept in CO^2^ incubator overnight for adherence to the plates. Next day, cells were irradiated with even doses from 2Gy to 8Gy and incubated at 37°C for colony formation. After 14 days, colonies were fixed with absolute ethanol and stained with 0.1% crystal violet. Colonies consisting of 50 or more cells were counted as clonogenic survivors. The percent plating efficiency, D0 value (radiation dose at which 37% population survives) and surviving fraction at a given radiation dose were calculated on the basis of survival of non-irradiated cells as described earlier [40]. Three independent experiments were performed, each time in duplicates with parental and radioresistant sublines and cell survival curve was plotted after calculating surviving fraction at each dose. Further, One-way ANOVA statistical analysis was performed to find the significant difference in survival at different doses of radiation.

**Figure 4:**
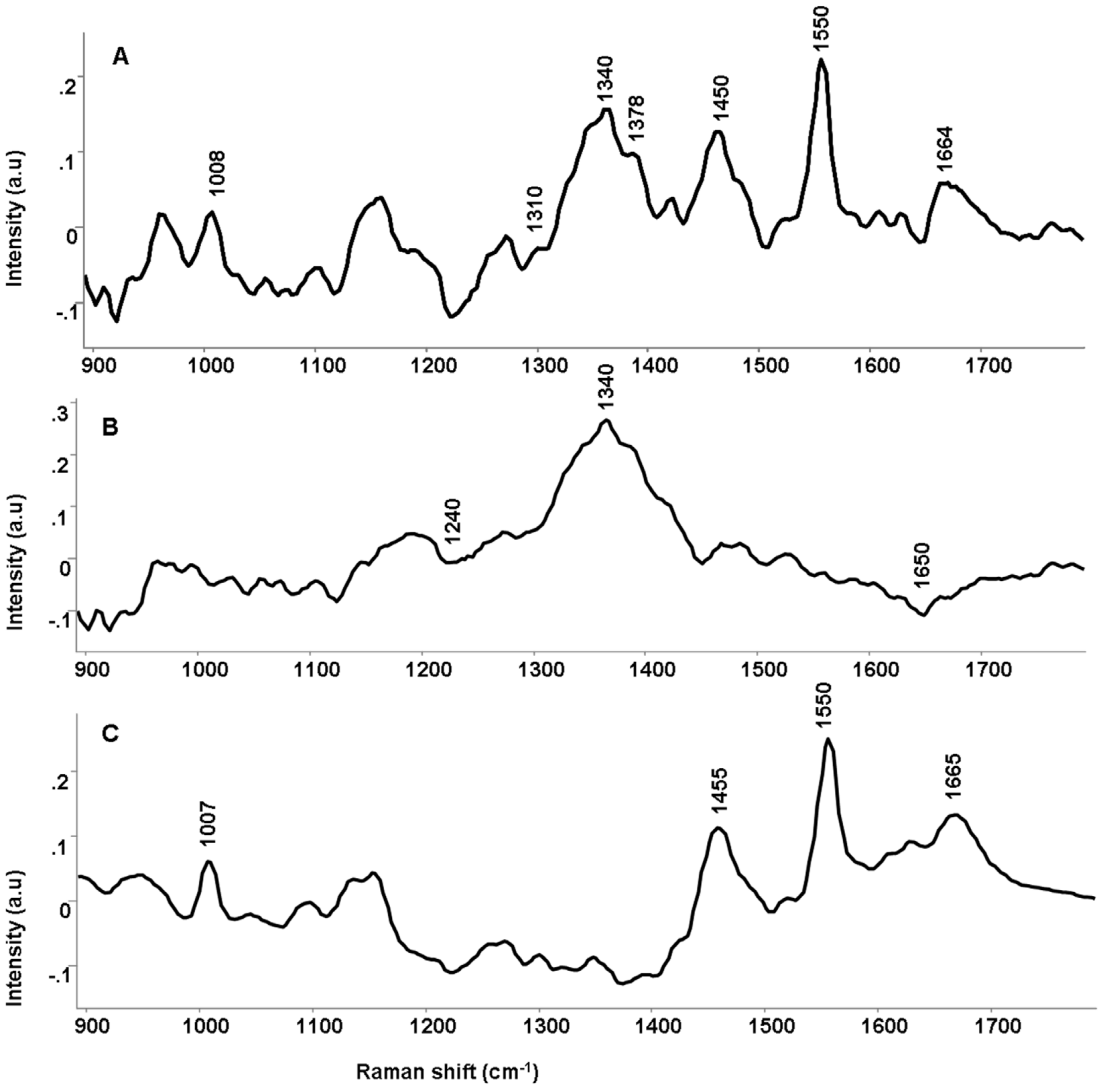
Difference Raman spectra of parental and radioresistant sublines. (A) 50Gy-UPCI:SCC029B subline – parental UPCI:SCC029B cell line (B) 70Gy-UPCI:SCC029B subline – parental UPCI:SCC029B cell line (C) 50Gy-UPCI:SCC029B subline –70Gy-UPCI:SCC029B subline.

**Figure 5:**
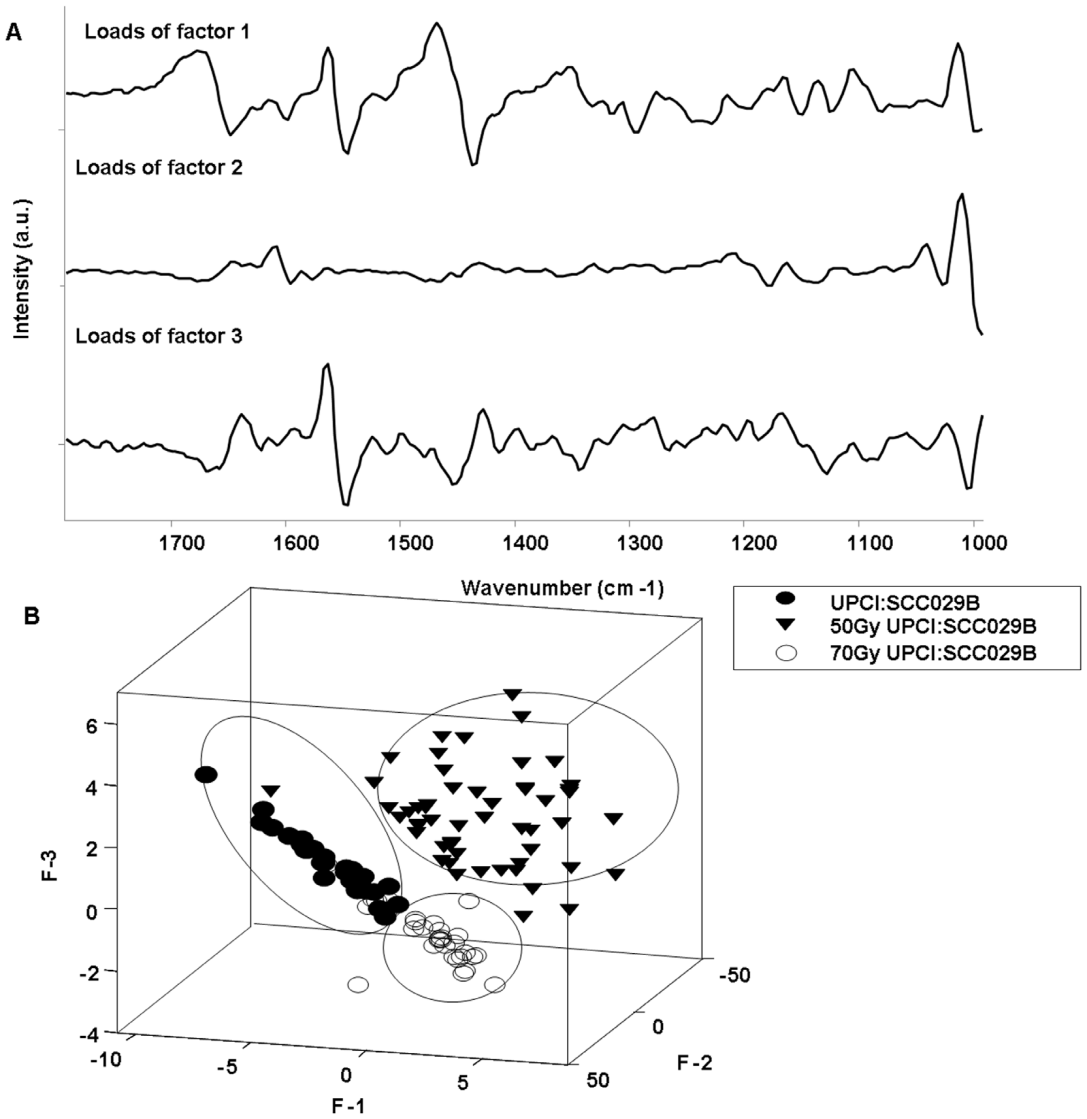
PCA analysis for parental and radioresistant sublines. (A) Loads of factor 1, 2 and 3 (B) 3-D scatter plot for parental UPCI:SCC029B cell line, radioresistant 50Gy-UPCI:SCC029B and 70Gy-UPCI:SCC029B sublines.

#### c) Western blotting

Cells were lysed in mammalian cell lysis buffer containing 1% protease inhibitor (Thermo scientific, USA). The cell lysate was centrifuged at 13,000 rpm for 10 min at 4°C and supernatant containing total cellular protein was collected. The protein concentration was quantified by colorimetric assay [41]. Samples containing 40 µg total proteins were separated by 12% SDS-PAGE and transferred to a PVDF membrane (PALL laboratory, USA). The membranes were blocked at room temperature for 1 hour by incubation in TBS containing 0.1% Tween (TBST, pH 7.4) and 5% (w/v) low fat milk. After blocking, membranes were incubated with rabbit polyclonal IgG human anti-Mcl-1 (1∶1000, sc-20679); Bcl-2 (1∶500, sc-492), Survivin (1∶1000, sc-10811), goat polyclonal IgG anti-Cox-2 (1∶500, sc-1747) and housekeeping rabbit polyclonal IgG anti-β Actin (1∶3000, sc-1616); (Santa Cruz Biotech., USA) overnight in blocking buffer. After washing six times in TBST, the membranes were incubated with an HRP-conjugated anti-rabbit IgG antibody (1∶2800) or anti-goat IgG antibody (1∶2500, Santa Cruz Biotechnology) in blocking buffer for 1 hour. After washing six times in TBST and two times in TBS, primary antibody binding was visualized by enhanced chemiluminescence substrate system (GE healthcare, USA). The western blotting was performed on three independent cell lysates of parental, 50Gy and 70Gy cells. The densitometry analysis was performed by Image J software (NIH, USA) against β-Actin housekeeping protein expression.

#### d) Morphological evaluation and F-Actin staining

Morphological changes observed during fractionated ionizing radiation were photographed by using the inverted microscope (Axiovert-200M, Zeiss) coupled with digital camera. Representative images of parental UPCI:SCC029B cell line, 50Gy-UPCI:SCC029B and 70Gy-UPCI:SCC029B sublines were processed by using Axiovision software (release 4.7, Carl Zeiss).

For filamentous Actin staining, cells were grown on coverslips, fixed with paraformaldehyde (4%) for 15 minutes and permeabilized in 0.7% Triton-X. A high affinity filamentous Actin (F-Actin) probe Alexa Fluor-488 phalloidin (Life technologies, USA) was diluted 1∶20 (in 1X PBS) and incubated with cells on coverslips for 30 minutes at room temperature in dark. After incubation, the coverslips were washed two times with 1X PBS for 10 minutes. DAPI (1∶20 diluted in 1X PBS) staining was done for approximately 1 minute and coverslips were then mounted in anti-quenching mounting agent (Vectashield, Vector labs, USA) on a clean glass slide and examined with LSM 510 Meta Carl Zeiss confocal system (Carl Zeiss Micro Imaging GmbH, Germany). Each of the parental, 50Gy and 70Gy cells were grown in duplicates on coverslips and random images for 50 cells were acquired. In this way, the staining was performed three times independently and 50 cells were analysed each time from all the cell population types for filopodia counting.

### Raman Spectroscopy

#### a) Sample preparation and spectral acquisition

Parental UPCI:SCC029B, 50Gy-UPCI:SCC029B and 70Gy-UPCI:SCC029B cells were cultured in 100 mm culture plates. Exponentially growing cells (3×10^6^ cells) from 6 independent cultures of each of the parental, 50Gy and 70Gy cells were harvested and phosphate buffer saline (PBS) wash was given to the cell pellets prior to spectra recording. Approximately 7 spectra were acquired from each cell pellet by using fibre-optic Raman microprobe system as described earlier [33]. Thus a total of ∼ 40 spectra per group were acquired for each of the parental, 50Gy and 70Gy cells.

As mentioned above, Raman system utilized for study consists of a diode laser (Process Instruments) of 785 nm wavelength as excitation source and a high efficiency (HE-785, Horiba-Jobin-Yvon, France) spectrograph coupled with a CCD (Synapse, Horiba-Jobin-Yvon, France) as detection element. Optical filtering of unwanted noise including Rayleigh signals are accomplished through ‘Superhead’ the auxiliary component of the system. Super head coupled with a 40× microscopic objective (Nikon, NA 0.65) was used to deliver laser light as well as to collect Raman signals. The spectrograph has no movable parts with fixed 950 gr/mm grating and spectral resolution as per manufacturer’s specification is ∼ 4 cm^−1^. Estimated laser spot size at the cell pellet sample was 5–10 µm. Spectra were integrated for 6 seconds and averaged over 3 accumulations. Typical laser power at the specimen was 40+0.05 mW.

#### b) Spectral pre-processing and data analysis

Raman spectra were pre-processed by correcting charged couple device (CCD) response by a National Institute of Standards and Technology (NIST) certified standard reference material 2241 (SRM 2241) followed by subtraction of background signals from optical elements and CaF_2_ window. To remove interference of the slow moving background, first derivatives of spectra (Savitzky - Golay method) were used for data analysis [42], [43]. Then spectra were interpolated in the 900–1800 cm^−1^ range and vector normalized. Analysis of the pre-processed spectra was carried out using PCA (principal component analysis) algorithms implemented in MATLAB (Mathwork Inc.) based in-house software [44]. PCA is unsupervised data overview tool used to look at the differences and similarities among the spectra. This method reveals outliers, groups and trends in the data. It describes data variance by identifying a new set of orthogonal features, these are known as loads of factor or principal components (PCs) (p<0.05). Principal components are linear combinations of original data variables. As the present study is an exploratory study therefore we have carried out PCA method of analysis and also used this method in our earlier *in vitro* studies on cell lines [35], [36].

Mean and difference spectra were calculated for different cell populations and were used for spectral comparison. The mean spectra were computed from the background subtracted spectra prior to derivatization by averaging Y-axis variations and keeping the X-axis constant for each class. The baseline correction was performed by fitting a 5^th^ order polynomial function. These baseline corrected spectra were used for spectral comparisons across all groups. Difference spectra were generated by subtracting the mean spectra of radioresistant cells (50Gy-UPCI:SCC029B & 70Gy-UPCI:SCC029B) with parental cells (UPCI:SCC029B) as well as by subtracting spectra of 50Gy-UPCI:SCC029B from 70Gy-UPCI:SCC029B cells.

### Statistical Analysis

Data were statistically analysed by One-way ANOVA and Student’s t-test, using Graph Pad Prism 5 software (version 5.01). A p value less than 0.05 was considered statistically significant. The statistical analysis used for Raman spectra processing are described under the respective section.

## Results and Discussion

### a) Development and Validation of Radioresistant Sublines

The radiotherapy protocols for oral cancer treatment consist of a total 50Gy to 70Gy radiation dose vide low dose fractionated radiation of 2Gy. Hence, two radioresistant sublines i.e. 50Gy-UPCI:SCC029B and 70Gy-UPCI:SCC029B were established by total 25 and 35 fractions of 2Gy respectively over a period of 5–6 months. The radioresistant character of sublines was demonstrated by clonogenic cell survival assay. We have observed significant increase in cell survival for both of the radioresistant sublines as compared to its parental cell line by clonogenic assay [Fig-1(A)]. D0 doses for the parental, 50Gy and 70Gy subline were calculated and found to be 4.5Gy, 5.1Gy and 5.6Gy respectively. An increase in the D0 value for the radioresistant sublines from its parental cell line indicates their acquired radioresistance character. We also determined the status of known radioresistant and anti-apoptotic protein markers like Bcl-2 (B cell lymphoma-2), Mcl-1 (myeloid cell leukemia-1), Survivin & Cox-2 (cyclooxygenase-2) by western blotting. As illustrated in Fig-1(B) an increase in the levels of these proteins was observed in radioresistant sublines (except Mcl-1 in 50Gy-UPCI:SCC029B) as compared to parental cell line. The Mcl-1 levels in the intermediate 50Gy-UPCI:SCC029B subline were although comparable (densitometry analysis, Figure-1B, lower) to that of parental cell line and was found to be further significantly upregulated in the radioresistant 70Gy:UPCI-SCC029B subline. It is noteworthy that Mcl-1 has short half-life due to its rapid turnover through ubiquitination [45] and although Mcl-1 cellular stability against various stress factors has been studied but little is known about its regulatory mechanism on account of radiation treatment. Our result possibly implies that pathways including Bcl-2, Survivin and Cox-2 may contribute to the increased survival for radioresistant cells at early stages of acquired radioresistance development while Mcl-1 may have a role in later stage. Moreover, as mentioned in clonogenic cell survival assay, the 50Gy-UPCI:SCC029B cells acquired the radioresistance character and higher levels of other radioresistance related proteins in comparison to the parental cells. Therefore, development of radioresistance is a complex phenomenon that cannot be associated with a single marker or protein within the cell. The significant increase in the expression of these markers corroborates with earlier reports, including those from our laboratory on their association with radioresistance in oral squamous cell carcinomas [13], [18]–[20], [46]–[47].

### b) Morphological Characterization of Radioresistant Sublines

We have observed altered morphology of radioresistant sublines in comparison to its parental cell line. The 50Gy-UPCI:SCC029B cells exhibited spindle shaped morphology while 70Gy-UPCI:SCC029B cells were found to be more elongated and irregular in shape [Fig-2(A)]. The gain of these morphological features in radioresistant sublines might hint towards its transformed characteristic related to the migration and invasion. Further, in order to get an insight in their actin reorganization; we have performed filamentous Actin (F-Actin) staining in the parental, 50Gy and 70Gy UPCI:SCC029B cells. F-Actin staining showed significant increase [Fig-2(B)] in the number of filopodia in 50Gy (p<0.01) and 70Gy-UPCI:SCC029B (p<0.001) cells in comparison to parental UPCI:SCC029B cells. The morphological changes exhibited by the radioresistant cells can be an additional phenotype acquired due to the continuous fractionated radiation treatment.

### c) Raman Spectroscopy of Parental and Radioresistant Sublines

The mean normalized spectra for parental UPCI:SCC029B cell line, radioresistant 50Gy and 70Gy-UPCI:SCC029B sublines were computed and depicted in Figure-3. Mean spectrum has been used as a representative spectrum for the respective cell lines in the spectral analysis. As, illustrated in Figure-3, the spectral features were dominated by bands around 1008 (phenylalanine), 1270 (amide III), 1450 (δ CH_2_) and 1660 (amide I) cm^−1^ and may be attributed to cellular proteins. Whereas, bands at 1310 (CH_3_/CH_2_) twisting or bending modes of lipid), 1340 (ring structure of adenine) and 1378 cm^−1^ (ring breathing modes of nucleic acid) suggest presence of cellular nucleic acid and lipids. The mean spectra for 50Gy-UPCI:SCC029B cells [Fig-3(B)] showed shift around 1310, 1450 and 1660 cm^−1^; while a prominent peak was observed at 1550 cm^−1^ (tryptophan). Similarly, in mean spectra of radioresistant 70Gy-UPCI:SCC029B cells [Fig-3(C)] intensity related variations at 1270, 1310, 1340 and 1660 cm^−1^ whereas a shift at 1450 cm^−1^ was observed. Thus, it can be observed that overall differences in the form of shifts in Raman bands and intensity variations were observed in the average spectra of both the 50Gy and 70Gy groups.

In order to bring out spectral differences among groups; the difference spectra were calculated from mean spectra. The difference spectra provide a more clear illustration for the differences among groups. As shown in Figure-4, the difference spectra were computed for 50Gy-UPCI:SCC029B and 70Gy-UPCI:SCC029B cells by subtracting them from parental UPCI:SCC029B cells [Fig-4(A, B)] and between the two radioresistant cells [Fig-4 (C)]. The difference spectrum (50Gy - parental) exhibited positive peaks at 1008, 1340, 1378, 1450, 1550, 1664 cm^−1^ and negative peaks at 1240 and 1310 cm^−1^; suggesting changes in proteins and nucleic acids, possibly due to altered cell signalling cascades caused by irradiation [48]. The noticeable change at 1550 cm^−1^ in 50Gy cells is indicative of freely accessible tryptophan possibly as a result of protein folding/misfolding by activated chaperones due to radiation stress. Similarly, in difference spectrum (70Gy - parental) positive peaks were observed at 1340 and 1378 cm^−1^ which is associated with DNA, whereas negative peak were observed at 1240, 1450 and 1650 cm^−1^ associated with protein change.

As shown by clonogenic assay, both the 50Gy-UPCI:SCC029B and 70Gy-UPCI:SCC029B cells have acquired radioresistant character in comparison to parental cells. Also, 70Gy cells are relatively more radioresistant than 50Gy cells and exhibit distinct difference spectra, which may be due to different properties acquired by them. Moreover, the (50Gy-70Gy) difference spectrum show positive peaks at 1008, 1450, 1550 and 1664 cm^−1^ all related to proteins while DNA related negative peak was observed at 1378 cm^−1^. The presence of prominent positive tryptophan peak (1550cm^−1^) in 50Gy radioresistant subline hint towards the enriched tryptophan moieties that may have anti-oxidative property because of the indolic group which serves as hydrogen radical donor [49]. Since, ionizing radiation can induce reactive oxygen species (ROS) production that can cause endogenous attack on the deoxyribosyl backbone of DNA [50], [51]. To counteract the effect of ROS, cells have several antioxidant factors that can scavenge ROS and protect against radiation [52]. The distinct tryptophan residue peak in 50Gy radioresistant cells might correlate with such factors that are rich in these residue types which shields the cell against oxidative stress. The upregulation of these factors have also been reported in the context of radioresistance [53]–[55]. Moreover, the recorded spectrum may result in an average and representative spectrum of the given cell line that reflects an overall information of the cell status; because in a cell pellet, the probing beam encounters a stack of cells and scattering can be expected from different organelles like nucleus, mitochondria and other cellular compartments.

### d) Multivariate Analysis

As mentioned above, PCA was used to explore the feasibility of classification among radioresistant 50Gy and 70Gy sublines from the parental cell line. PCA is frequently used method for data compression and visualization to observe the pattern in the data. It is a mathematical analysis by which the features in the whole data set of thousands of points are resolved into a few significant eigenvectors that can express the entire data set with their scores for each spectrum. This can provide imperative clues on biochemical variations among different groups, in our case different classes of macromolecules. Further, the profiles of principal components also known as factor loadings can provide vital clues on biochemical variations among different classes. Loading of factors 1, 2 and 3 that lead to demarcation among groups are presented in Figure-5(A). Conforming spectral variability as suggested by difference spectra; the loading plots also indicate differences in DNA content, amino acids and protein profiles of parental and radioresistant groups. For visual discrimination, we project each of the spectra in the newly formed co-ordinate space of selected PCs. First three significant discriminating PCs were selected for three-dimensional visualization of the data. Three clusters belonging to parental UPCI:SCC029B cells and radioresistant 50Gy- UPCI:SCC029B, 70Gy-UPCI:SCC029B cells was observed [Fig-5(B)]. While 50Gy-UPCI:SCC029B cells formed a separate cluster but slight overlap between the clusters of 70Gy-UPCI:SCC029B and parental cells was observed. These pattern clustering may be due to overall different biochemical profile acquired by the cell types. Taking in view that PCA will reveal an overall change including the various cellular profiles, it indicates that radioresistant cells have acquired an altered molecular profile different from its parental cells with subtle variations.

## Conclusion

In the present work, we have established radioresistant sublines from the parental oral buccal mucosa cell line using clinically admissible fractionated radiation dose. The acquired resistant character was determined by the standard clonogenic cell survival assay. The 50Gy-UPCI:SCC029B and 70Gy-UPCI:SCC029B established radioresistant sublines were found to be more radioresistant in comparison to its parental UPCI:SCC029B cell line. The sublines were also characterized by assessing expression of radioresistance related protein markers like Mcl-1, Bcl-2, Cox-2 and Survivin that support their acquired radioresistant phenotype. Altered morphological features were observed in these long term irradiated cells that were different from the parental cells, including significant increase in filopodia numbers in 50Gy-UPCI:SCC029B cells and 70Gy-UPCI:SCC029B radioresistant cells. Further, Raman spectroscopy was performed on these radioresistant cells and parental cells to study their differential spectral profile. This study is first of its kind regarding utility of RS in characterization of acquired radioresistant sublines. The observed differential spectra between parental and both the radioresistant cells were majorly due to changes in DNA, lipid and protein profile of cells. Alterations in DNA content of these cells may be because of numerous genetic insults occurring through multiple fractions of radiation by FIR and change in lipids may be predominantly due to the altered morphology of these cells as shown above. The multivariate analysis using PCA revealed that the radioresistant 50Gy-UPCI:SCC029B and 70Gy-UPCI:SCC029B cells can be categorized from its parental UPCI:SCC029B cells. Taken together, the results of our work are quite promising and suggest the feasibility of RS as a potential non-invasive tool for oral cancer patients in predicting radiation response through spectral markers. It may improve the patient survival rates by virtue of optical diagnosis to categorize them in radiosensitive and resistant types; thereby help in selecting better treatment regimens.
